# Pre-amyloid oligomers budding:a metastatic mechanism of proteotoxicity

**DOI:** 10.1038/srep35865

**Published:** 2016-10-24

**Authors:** Fabrizio Bernini, Daniele Malferrari, Marcello Pignataro, Carlo Augusto Bortolotti, Giulia Di Rocco, Lidia Lancellotti, Maria Franca Brigatti, Rakez Kayed, Marco Borsari, Federica del Monte, Elena Castellini

**Affiliations:** 1 Department of Chemical and Geological Sciences, University of Modena & Reggio Emilia, Modena, Italy; 2 Cardiovascular Institute, BIDMC, Boston, MA, 02215, USA; 3 Department of Neurology, UTMD School of Medicine, Galveston, TX, USA.

## Abstract

The pathological hallmark of misfolded protein diseases and aging is the accumulation of proteotoxic aggregates. However, the mechanisms of proteotoxicity and the dynamic changes in fiber formation and dissemination remain unclear, preventing a cure. Here we adopted a reductionist approach and used atomic force microscopy to define the temporal and spatial changes of amyloid aggregates, their modes of dissemination and the biochemical changes that may influence their growth. We show that pre-amyloid oligomers (PAO) mature to form linear and circular protofibrils, and amyloid fibers, and those can break reforming PAO that can migrate invading neighbor structures. Simulating the effect of immunotherapy modifies the dynamics of PAO formation. Anti-fibers as well as anti-PAO antibodies fragment the amyloid fibers, however the fragmentation using anti-fibers antibodies favored the migration of PAO. In conclusion, we provide evidence for the mechanisms of misfolded protein maturation and propagation and the effects of interventions on the resolution and dissemination of amyloid pathology.

Diseases of protein folding are growing age-dependent plagues worldwide, and the recognition of their combinatory triggers and potential coexistence^1^,^2^ is an alarming forthcoming, with the aging of the population. Despite the heterogeneity in terms of clinical features as well as time of onset and tissue distribution, age-related disorders share a specific pathological signature that is the accumulation of protein aggregates, forming amyloid fibers, that drive tissue damage and organ failure. Whether causative or epiphenomenon, malfolded (normal) or misfolded (abnormal/mutated) protein aggregation and accumulation has been shown to be an early event in the pathogenesis of proteinopathies and, importantly, to cause cell damage and death, disease development and progression^3^,^4^,^5^. Since the pathogenic mechanisms at the origin and progression of these diseases are unclear, there are no effective treatments, and a cure is lacking. Major obstacles in the therapeutic approach to proteinopathies include the identification of the toxic amyloid-related entities, the still unclear nature of the mechanisms of proteotoxicity, and the dynamic changes in the process of amyloid fiber formation and dissemination.

In this study we begun addressing some of these unknown using an *in-vitro* reductionist approach by atomic force microscopy (AFM) to study the dynamic events of amyloid de/polymerization and migration. We aimed at defining the temporal and spatial dynamic changes of misfolded protein aggregation and fibrillogenesis, and their modes of dissemination. This is important because identifying how amyloid develops and the steps from misfolded proteins to fibrillar formation, expansion and metastasis will allow tailoring our interventions to each of the different maturation and propagation stages. The ultimate goal would be to interfere with the key mechanisms responsible for the development, progression and/or exacerbation of the organs damage and block them.

## Results

### PAO growth *in situ* to large ordered amyloid fibers

**Figure 1:**
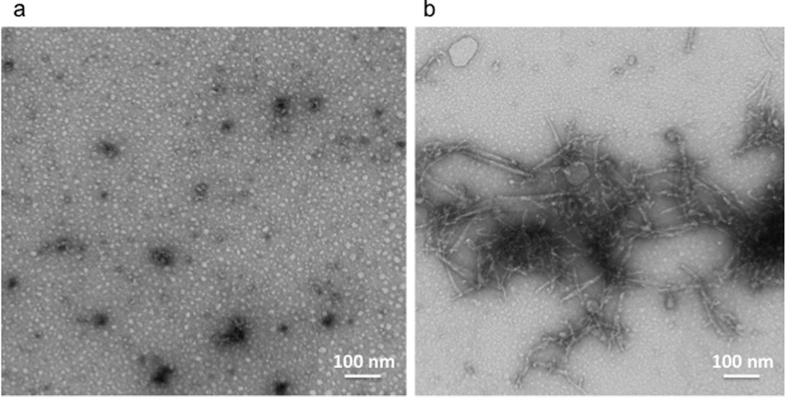
PAO and amyloid fibers trasmission electron micrographs of: (**a**) the PAO synthesized *in-vitro*; (**b**) the amyloid fibers formed after addition of a 5 v/v% hexane solution to the PAO in suspension.

**Figure 2:**
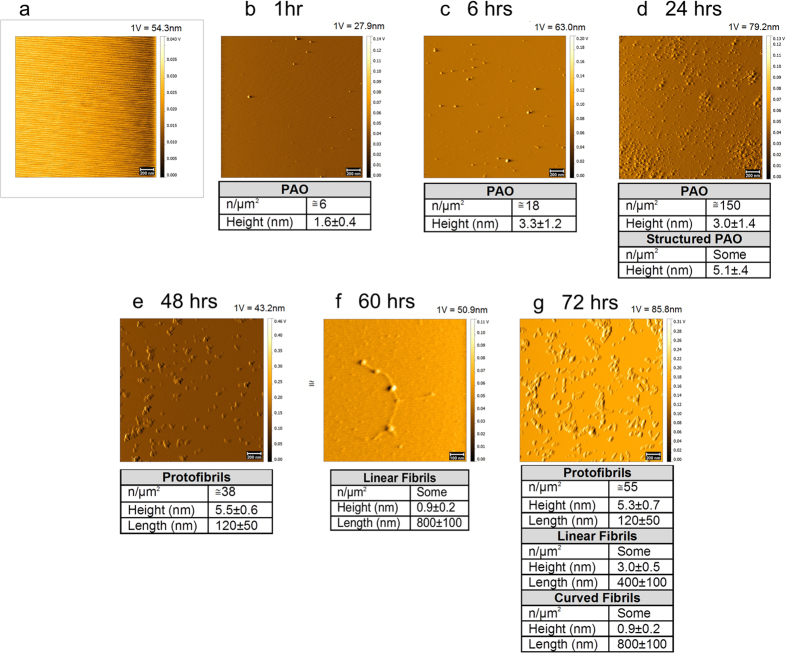
PAO nucleation on primary mica surface. PAO adsorbed on primary (Iry) mica surface followed over time. Nucleation of PAO leads to the formation of linear protofibrils after 48 hrs (**e**). Small fibrils were also observed after 60 hrs (**f**) and at 72 hrs (**g**). Scale bars: 100 nm (**f**) and 200 nm (**a–e,g**). (**a**) Contact Mode (CM) topography image of starting bare mica. (**b–g**) Tapping Mode (TM) topography images of PAO (**b–d**) and protofibrils/fibrils (**e–g**) adsorbed on mica after different contact times (1–72 hrs) with a 44 μM Aβ_42_ peptide solution in 10 mM PBS at pH 7.4, T = 25 °C. The number and size of the structures are indicated in the table under each experimental time point. The amplitude scale is in Volt; we indicated the height (nm) corresponding to 1 Volt.

Misfolding of proteins is known to progress from monomeric structures to complex amyloid fibers through reversible maturation steps^6^,^7^,^8^ (Supplementary Fig. 1). Here we interrogated how the different misfolded species thrive. We prepared oligomeric seeds and amyloid fibers from purified Aβ_42_ peptides as previously described^9^ and verified by transmission electron microscopy (TEM) (Fig. 1). We immersed slices of mica surface into glass tubes containing PAO in suspension. At intervals from 1 to 72 hrs the surfaces were pulled out, washed at least three times with ddH_2_O, blow-dried with argon, placed on the AFM platform and promptly measured. This set of experiments confirmed that PAO could be detected on mica surfaces (Fig. 2). We followed the maturation of PAO over time. Tapping mode (TM) topography images showed, at 1-24 hrs, pseudo spherical PAO (and perhaps larger aggregates) that increased over time in number and size (Fig. 2b–d). At times longer than 24 hrs, we observed protofibrils and fibrils (Fig. 2e–g). Also, in this case, the number of the structures on mica increased with time. In particular, the maturation of PAO showed the formation of linear protofibrils (Fig. 2e) after 48 hrs and small fibrils after 60 hrs (Fig. 2f). These results indicate that small PAO may grow at the site where they first originate and progressively mature to coalesce into linear protofibrils^10^,^11^,^12^,^13^,^14^. Over time fibrillar aggregates may become the amyloid plaques.

### PAO budding as a mechanism of tissue invasion

**Figure 3:**
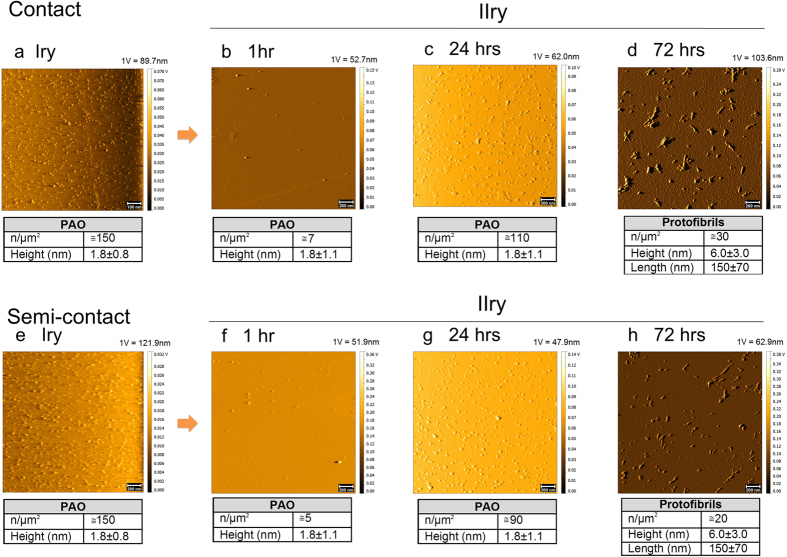
PAO migration. PAO adsorbed on a primary (Iry) mica surface were placed in contact as well in semi-contact with a secondary (IIry) surface. TM topography images of the PAO adsorbed on primary placed afterwards in contact (**a**) or in semi-contact (**e**) with a secondary mica surface and corresponding secondary mica at different times (1–72 hrs, contact **b–d**, semi-contact **f–h**). The images show the formation of PAO on the secondary surface after 1 hr of incubation. The number of PAO on the secondary mica is higher after 24 hrs of incubation and linear protofibrils appear after 72 hrs. Scale bars: 100 nm (**a**) and 200 nm (**b–h**). PAO were adsorbed on the primary mica by dipping the surface for 24 hrs in a 44 μM Aβ_42_ peptide solution in 10 mM PBS at pH 7.4, T = 25 °C. The number and size of the structures are indicated in the table under each experimental time point. The amplitude scale is in Volt; we indicated the height (nm) corresponding to 1 Volt.

Aβ_42_ fragments have been recently shown to be able to migrate to the brain *in-vivo* from a distant site of inoculation, acting as a prion disease^1^,^2^,^15^,^16^. Here we tested whether PAO can move from one surface to the other. We adsorbed PAO on a mica surface by immersion in the PAO-containing solution for 24 hrs (here referred as primary mica surface, Iry in the figures) (Fig. 3a,e). The surface was washed at least three times with ddH_2_O and placed in a glass tube containing phosphate buffered saline (PBS). Two sets of experiments were conducted. In a first set, a bare mica surface (here referred as secondary mica surface, IIry in the figures), without any treatment, was placed in direct contact with the primary surface, only separated by the remaining liquid film (hereafter in contact) (Supplementary Fig. 2). Based on the progression of the PAO accumulation of the first set of experiments (Fig. 2), we pulled out the secondary surface at 1, 24 and 72 hrs time intervals, washed at least three times with ddH_2_O, blow-dried with argon and imaged with AFM (Fig. 3b–d). We found that PAO were able to transfer between the two surfaces in contact as early as 1 hr. At 24 hrs PAO density increased and larger protofibrillar structures formed at 72 hrs (Fig. 3b–d).

In a second set of experiments a 0.1 mm thick Teflon thread was placed between the two plates to maintain them at a constant separation distance (hereafter in semi-contact). A set of primary and secondary surfaces was prepared as before and, at the same time intervals, the secondary surface was pulled out, washed, argon blow-dried and imaged with AFM (Fig. 3e–h). Similarly to what was observed without the Teflon thread, the secondary mica was coated by pseudo-spherical PAO at short contact times (1–24 hrs, Fig. 3f,g) and linear amyloid protofibrils were observed after 72 hrs (Fig. 3h). For both contact and semi-contact experiments, the height of PAO and the dimension of protofibrils are in agreement with previously reported data obtained by AFM investigations^17^,^18^,^19^.

These results showed that oligomers can be released from a surface where they strongly adhere and move away to attach to another distant surface. Thus these results, in combination with the observation of the maturation of PAO to fibers, may explain the dynamics of tissue invasion and spreading as well as the time lapse in the progression of the disease pathology.

### Dynamic fiber depolymerization and re-polymerization as a mechanism of tissue invasion

To better understand the spatio-temporal migration of PAO between surfaces we tested how mature fibers that are adherent on a surface can release PAO. We tested the concept that amyloid fibers, considered inert, may be indirectly toxic by releasing PAO and other misfolded aggregate species in the interstitial tissue poisoning neighbor cells.

**Figure 4:**
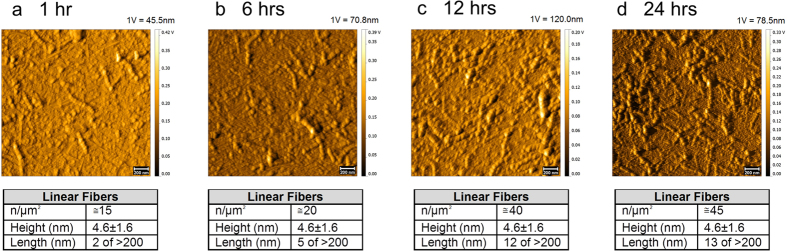
Fibril adsorption on primary mica surface. PAO were fibrillated and adsorbed on primary (Iry) mica and followed up to 24 hrs. Images show progressive growth of the number and size of the fibrils. Scale bars: 200 nm. PAO were fibrillated as reported in the Methods section. TM topography images show progressive growth of the number and size of the fibrils. At all the investigated times, the length of the fibril varies from 80 to 700 nm. The number and size of the structures are indicated in the table under each experimental time point. The amplitude scale is in Volt; we indicated the height (nm) corresponding to 1 Volt.

We fibrillated the oligomer preparation with hexane as previously described^9^ and adsorbed the fibrils on a primary mica surface, as for the PAO, for 1, 6, 12 and 24 hrs to identify the optimal time to visualize the fibrils immobilized on the mica surface and characterize the morphology of the resulting surface (Fig. 4). The results indicated an increase in fibril density and length, but not height as a function of time and the appearance of well-defined fibers at 24 hrs. This suggests that the adsorption of pre-existing fibrils is the main mechanism involved in the aggregation process on the mica surface. Therefore, since the size of fibers remained constant with a high surface density we selected 24 hrs as the time point to adsorb the fibrils on the primary plate for the subsequent experiments.

**Figure 5:**
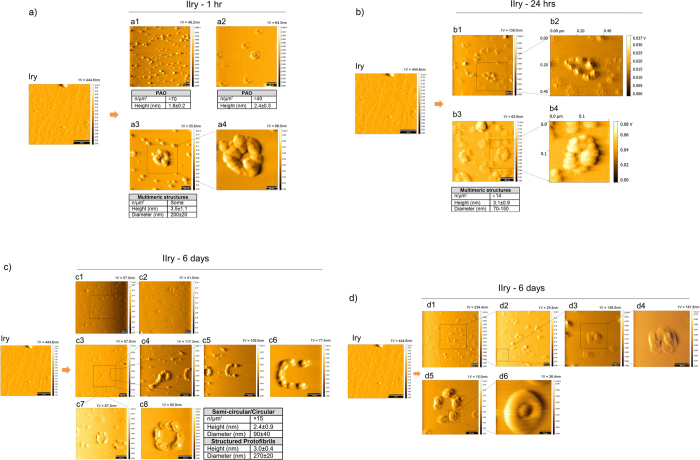
PAO budding and migration. Fibrils were adsorbed on primary (Iry) mica surface (scale bar 1 μm). An empty secondary (IIry) mica was placed in contact with the primary mica and followed up to 6 days. Contact solution: 10 mM PBS at pH 7.4, T = 25 °C. A TM representative topography image of a pristine primary coated with fibrils is shown as primary (Iry). (**a**) Numerous PAO are adsorbed on the secondary mica after 1 hr of contact (scale bars: 50 nm (a2,a4) and 100 nm (a1,a3). At this time transitional structures (a2) and scarce annular protofibrils (a3–a4) are adsorbed on the IIry. (**b**) More complex multimeric structures (MS) appear after 24 hrs (scale bars: 100 nm). The inserts b2 and b4 were magnified with the software therefore do not have the scale bar within the figure. (**c,d**) semi circular, circular (c1–c8) and more structured protofibrils (c4) and transitional structures (d5,d6), appear after 6 days (scale bars: 10 nm (d6), 50 nm (c6,c8,d3–d5), 100 nm (c2,c4,c5,c7,d2), and 200 nm (c1,c3,d1)). The number and size of the structures are indicated in the table under each experimental time point. The amplitude scale is in Volt; we indicated the height (nm) corresponding to 1 Volt.

**Figure 6:**
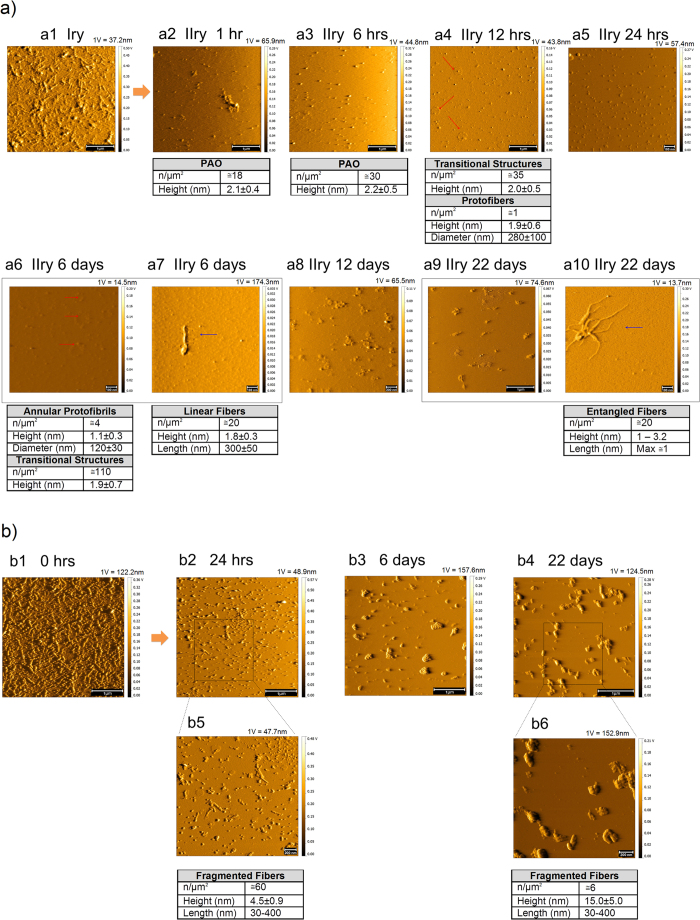
PAO budding and metastasis. **(a)** Fibrils were adsorbed on primary (Iry) surface (scale bar: 1 μm). Empty secondary (IIry) mica was placed in semi-contact with the primary mica and followed up to 22 days. Contact solution: 10 mM PBS at pH 7.4, T = 25 °C. (a1,b1) show the TM topography image of a pristine primary coated with fibrils. Here also numerous PAO appear on the secondary surface after 1 hr whose number increases at 6 hrs. Complex structures (PAO aggregates/transitional structures and annular protofibrils – red arrows) appear as soon as 12 hrs growing in number and complexity at later time points. Scarce simple fibrils (blue arrow) appear after 6 days and more complex fibrillar structures (blue arrow) after 22 days (scale bars: 100 nm (a5–a7,a10), 200 nm (a8), and 1 μm (a2–a4, a9)). (**b**) TM topography images of the structure present on the primary after semi-contact at different times (1–22 days), the images b2–b6 show a progressive fragmentation and reorganization of fibers with time (scale bars: 200 nm (b5,b6) and 1 μm (b2–b4)). The number and size of the structures are indicated in the table under each experimental time point. The amplitude scale is in Volt; we indicated the height (nm) corresponding to 1 Volt.

After 24 hrs of exposure, the fibrillar-containing surface was washed and placed in a glass tube containing PBS as described above. Fibril migration experiments were performed, with the bare secondary plate put in contact (Fig. 5) or semi-contact (Fig. 6) with the primary.

Here we showed that immobilized fibrils are indeed dynamic structures that can release PAO. From the primary surface oligomers were budding and deposit on the secondary surface whether the two surfaces were in direct contact (Fig. 5) with the primary or physically separated by 0.1 mm of physiological solution filled space (Fig. 6). In both experimental conditions, we observed PAO maturation on the secondary plate over time. Transitional structures and some annular protofibrils were formed on the secondary surface in direct contact with the primary, as early as 1 hr of exposure (Fig. 5a,a1–a4). At 24 hrs we observed numerous multimeric structures (Fig. 5b,b1–b4). After 6 days of exposure we could observe the appearance of semicircular and circular fibrils and aggregates (Fig. 5c,d).

When the secondary plate was in semi-contact we observed numerous PAO whose number increased between 1 hr and 6 hrs (Fig. 6a2,a3) and the appearance of transitional and annular structures after 12 hrs of exposure (Fig. 6a4 red arrows). Scarce fibrils were also detectable as early as 6 days and more entangled fibers appeared after 22 days of exposure (Fig. 6a9,a10 blue arrows). Fibrillar structures deplete from the primary plate over time (Fig. 6b1–b6). It is possible that fibrils progressively broke to form the PAO fragments that migrated to the secondary surface leaving smaller and less organized protofibrils behind. Those, in turn, may regain higher toxic activity. The experiments performed in contact and semi-contact differ not only in the migration rate to the secondary, but also in number and dimensions of adsorbed species. This suggests that the distance (diffusion time) between the depolymerization and repolymerization sites partially affects the nature of the deposited species.

### Antibodies treatment affects the rate of formation, maturation and metastasis of misfolded proteins

**Figure 7:**
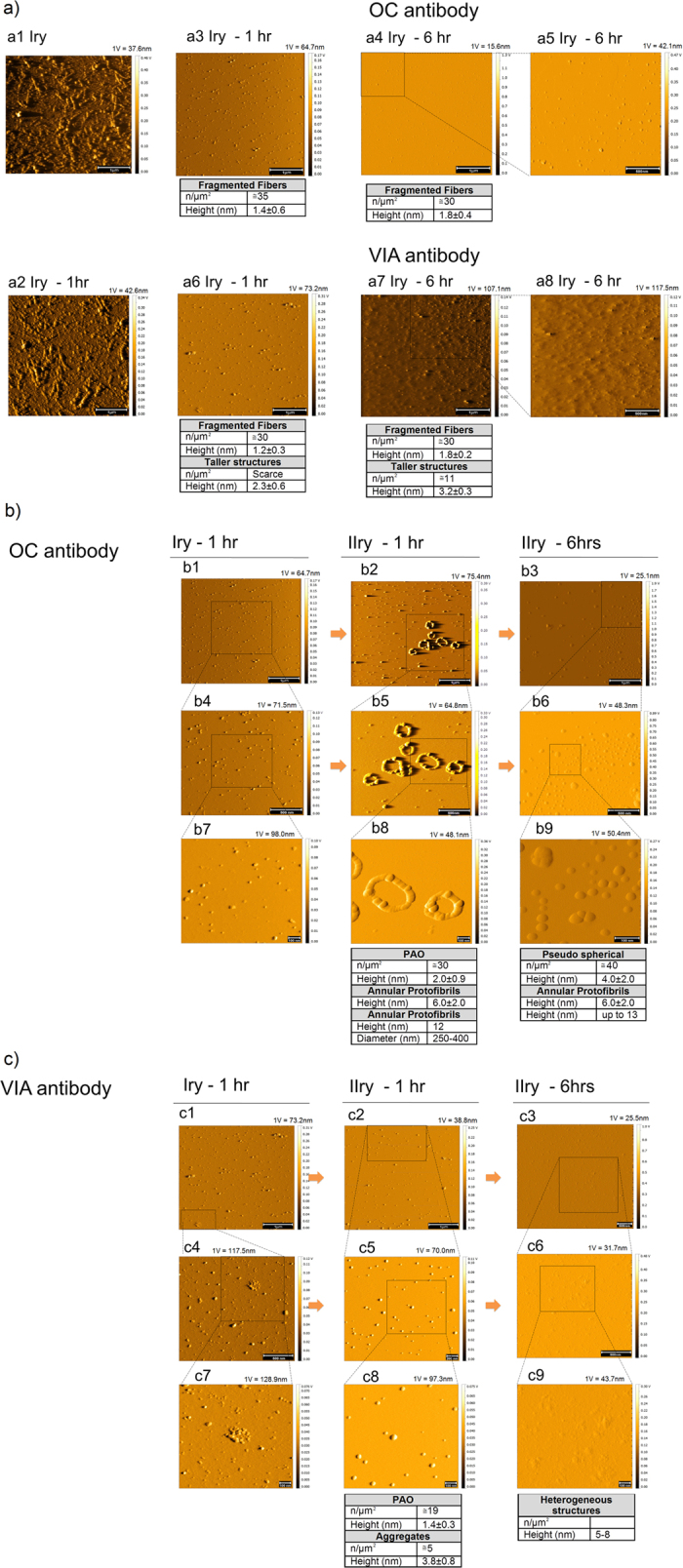
Fiber and PAO response to structural antibodies. Treatment with antibodies against amyloid fibers (OC) and Aβ_42_-PAO (VIA) results in fragmentation of the fibers on the primary surface **(a)** while PAO and circular protofibrils grow on the secondary surface with the anti-fiber antibodies (OC) **(b)**. Treatment with antibodies against Aβ_42_ PAO (VIA) also led to fibers fragmentation both on primary and secondary surfaces **(c).** (Scale bars: 100 nm (b7–b9,c7–c9), 200 nm (c5), 500 nm (a5,a8,b4–b6,c3,c4,c6), and 1 μm (a1–a4,a6,a7,b1–b3,c1,c2)). **(a)** TM topography images of fibrils adsorbed on a primary and placed in semi-contact for 0, 1 and 6 hrs with a secondary using solutions containing structural ≅5 μM OC or VIA antibodies. The image of a pristine primary coated with fibrils is shown in a1. For comparison, a2 shows the same surface after 1 hr of semi-contact in 10 mM PBS alone. (**b,c**) TM topography images of a primary after 1 hr and the corresponding secondary after 1 hr and 6 hrs using solutions containing OC (**b**) and VIA (**c**). For both antibodies, pseudo-spherical fibril fragments are observed on the primary (a3–a8). After 1 hr of semi-contact with OC antibodies (b2,b5,b8), PAO and annular protofibrils grow on the secondary; later (6 hrs, b3,b6,b9) non homogenously dispersed pseudo-spherical PAO and aggregates of large dimensions are observed. After 1 hr of semi-contact with VIA antibodies (c2,c5,c8) spherical PAO and aggregates form on the secondary, later (6 hrs, c3,c6,c9) a deposition of heterogeneous structures arises, some of which reaching the height of 5–8 nm. The number and size of the structures are indicated in the table under each experimental time point. The amplitude scale is in Volt; we indicated the height (nm) corresponding to 1 Volt.

Immunotherapy against Aβ deposits has been recently applied to prevent aggregation or accelerate removal of amyloid plaques in the brain. Whereas removing the pathological accumulation of amyloid fibers would per se provide a legitimate therapeutic rationale, the concept that PAO fragments, rather than fibrillar deposition, are the toxic species, raises the question of whether fibrillar dissolution would favor the fragmentation into and the diffusion of the more toxic PAO. In fact, passive immunotherapy using monoclonal antibodies that target the mid-domain of Aβ peptide recognizing the soluble non-fibrillar form of Aβ has demonstrated improvement of cognitive function in patients with mild AD^20^. Instead monoclonal antibodies targeting epitopes on fibrillar Aβ, disassembling amyloid plaques by activating phagocytosis, failed to show efficacy compared to placebo^21^. Given the dynamic equilibrium between amyloid fibers, intermediate species and PAO and the result of our data on fibrillar fragmentation into PAO, we sought at testing whether promoting the dissolution of Aβ fibers would increase PAO formation and metastasis. Thus, we repeated the experiments depicted in Fig. 6 using primary mica with fibers in semi-contact in the presence of anti-fibrils antibodies (OC^22^) or anti-Aβ_42_PAO antibodies recognizing the last three amino acids of the Aβ_42_ (Val-40, Ile-41, Ala-42 = VIA^23^). Our results showed that the effect of OC antibodies is to fragment the fibrils deposited on the primary mica surface (Fig. 7a1,a3–a5), but at the same times it promoted the migration of PAO and the formation of annular protofibrils on the secondary mica surface (Fig. 7b). The treatment with VIA antibodies, instead, resulted in fragmentation of the fibers and pulverization of the fragments on the primary mica (Fig. 7a2. On the secondary mica after 1 hr we observed a limited deposition of oligomeric structures, which undergo fragmentation and pulverization already after 6 hours (Fig. 7c), as observed for the primary mica surface.

As control, we imaged mica surfaces immersed in the solution containing OC and VIA antibodies only (Supplementary Fig. 3). These experiments showed that OC and VIA antibodies have low affinity for bare mica. The imaged antibodies are approximately one tenth for OC and a half for VIA of the surface concentration of species detected on secondary mica in the above experiments. This indicates that the adsorbed species on the secondary surface are predominantly deriving from the fibers fragmentation and not ascribable to direct antibody adsorption. This is further confirmed by the comparison of the height profiles of the antibodies and PAO on secondary surface, which substantially differ.

## Discussion

Amyloid fibers are the larger ordered molecular structures built from repeat units (*mer*). They form from a multistep process by which malfolded (if normal) or misfolded (if mutated) proteins undergo progressive conformational changes through a process of nucleation-dependent polymerization^24^,^25^,^26^ (Supplementary Fig. 1). This was originally thought to be a biphasic event by which, when misfolded protein monomers reach a critical concentration, they precipitate into fibrils in a stable equilibrium. The introduction of atomic force microscopy demonstrated the presence of intermediate species, their dynamic formation from monomers and their disappearance as fibers are formed^6^,^7^,^8^.

A correlate to the dynamic conformation growth is the propagation of the lesion within the tissue, spreading the disease. Clinically, amyloid pathology is a spatially progressive process and recent evidence has put forward the hypothesis that misfolded proteins may be transmitted between neurons^27^,^28^, acting in a prion-like behavior in a process named “*template protein corruption*”^29^. In this process, misfolded proteins can bind to cognate molecules and corrupt their conformation, propagating the lesion^1^,^2^. Previous evidence also demonstrated that, like prion, Aβ_42_ aggregates could propagate from peripheral inoculation in the peritoneal cavity causing β-amyloidosis to the brain^30^. Furthermore, very recently it was reported the potential for iatrogenic transmission, supporting the possibility that mal/misfolded fragments can maintain their toxic activity in a non-physiological ambient and spread from inert substrates to biological targets^15^,^16^.

Conform to these reports our data support the notion that aggregated structures can form in a relatively short time (hrs) from PAO and that those can progressively grow in number and complexity to more tangled structures. The aggregated structures can also be released from inert structures maintaining the potential to carry the pathogenic potentials. Furthermore, our findings reveal that PAO can migrate between structures in direct contact or at distance recreating and amplifying the pathology. A projection of our data to a physio-pathological setting would envision the possibility that PAO can bud from fibers released in the interstitial tissue from diseased cells by exocytosis or from dying cells. Those can then decorate the neighbor cells and re-trace the maturation steps to form fibers whether in direct contact or through fluid filled spaces. In fact, we also reveal that, although through longer time interval, the migration can occur even between structures physically separated. This may represent a mechanism for aggregates metastasis in tissues.

Understanding the mechanisms of maturation and spreading of the disease is important not only to learn how amyloid plaques, that begin more often in the hippocampus and entorhinal cortex, extend to most brain regions, but also to design effective and safe therapeutic approaches.

A still unclear aspect in the pathogenic mechanism of misfolded amyloid species is to which of these structures the toxicity is linked. The prevailing wisdom has been, for a while, that the insoluble amyloid fibers are responsible for the cellular toxicity. However, the extent of the plaques containing insoluble fibrils, did not correlate with the clinical development of the disease in Alzheimer’s dementia, whereas the soluble fraction composed of PAO correlates better with the symptoms of cognitive impairment^31^. This observation and recent experimental evidence has shown, in the neurons and cardiomyocytes, that, for the most part, the PAO and/or a mix of mono-trimers, oligomers and protofibrils carry the toxicity to the cell^9^,^32^. Thus it is critical to direct the therapeutic effort towards targeting the toxic entity e.g. by pushing the equilibrium against or towards fibrillogenesis or both.

The development of structural antibodies against fibers has been, in the recent years, viewed as a promising therapeutic approach to Alzheimer’s Disease in addition to the more traditional pharmacotherapy directed towards neurotransmitters receptors inhibition or activation or neurotransmitters metabolism. Immunotherapy against Aβ deposition was introduced in an effort to remove amyloid deposition. However, despite the reduction in amyloid plaques in the treated patients detected post-mortem, or by PET imaging in living patients, the development of meningoencephalic syndrome against a trivial clinical gain, called off clinical trials using immunization^33^. The rationale for immunotherapy against Aβ in fact mainly stems from *in-vitro* demonstration that antibodies against the N-terminal of the peptide prevent the formation and dissolve preformed fibrils and activate microglia to clear opsonized plaques^34^,^35^,^36^. Subsequently, animal studies indicated that active^37^ and passive immunization^38^ - even following a single injection^39^,^40^ - in transgenic mice models of AD could remove brain plaques. Differently, antibodies against the soluble Aβ - unable to bind fibrillar amyloid - were ineffective in dissolving amyloid plaques *in-vivo* and *in-vitro* in mice and to activate T-cell response^38^. However despite a residual plaque deposition, memory benefit could be achieved in these mice prompting the hypothesis that a complete removal of plaques was not necessary to obtain cognitive benefit^41^,^42^. Based on our results we put forward the hypothesis that the apparent contradicting correlation between the minimal resolution of plaques and the improvement of cognitive function results from targeting the toxic PAO. Thus we designed our experiments to test whether the immunization therapy would indeed improve amyloid pathology or, instead, potentially worsen the equilibrium towards the toxic species informing to which would be the best theoretical approach to improve disease pathology and, overall, the clinical outcome. Our results provide evidence that the antibody treatment of preformed amyloid fibers may not reach the desired therapeutic effect. This is because although on one end the treatment indeed reduces the plaques as observed in the clinical studies, at the same time, it would potentially increase the release of the toxic PAO nullifying the benefit acquired by dissolving the fibers. Long-term experiments are mandatory to test whether the increased PAO would metastasize and eventually worsen the fiber pathology with newly formed foci of amyloid plaques. Our experimental evidence points towards a potential benefit, instead, of immunotherapy using the anti-PAO antibodies that resulted in extensive fragmentation of the fibers. Thus this approach would on one end dissipate the fibers, but also probably dissolve the released PAO in smaller non-toxic fragments activating immunity and favoring removal. Long-term follow up experiments dedicated to understanding the fate of the fragments will clarify if a therapeutic approach with anti-PAO immunotherapy reduces plaques expansion and its dissemination, from the restricted prone regions, more extensively to the brain sectors and would bear benefit to the patients.

In conclusion, the dynamic processes, namely the nucleated polymerization and the prion-like propagation as well as the iatrogenic transmissibility contribute to the complexity of the pathogenesis of proteinopathies and of the therapeutic approaches. Understanding the mechanisms of protein aggregation and organ damage is critical to identify when and how to intervene in the different stages of the disease progression. It will also provide targets for therapeutic interventions for different progressive and deadly aging-associated diseases and aging itself.

## Methods

### PAO and fibril synthesis

1 mg lyophilized Aβ_42_ peptide (USBio) was used to prepare PAO as previously described^9^. The peptide was solubilized with acetonitrile and further dissolved by sonication. The peptide’s pH was neutralized with NaOH and phosphate buffer. A Phosphate-buffered saline (PBS) solution containing 0.02 w/v% NaN_3_ was then added to 1 mg lyophilized Aβ_42_ peptide (USBio) dissolved in acetonitrile to reach the final volume of 5 ml. After 20 min incubation at room temperature, the samples were centrifuged; the supernatant fraction was transferred to another tube and gently bubbled with an argon stream for 10 min to remove the acetonitrile. The complete removal of acetonitrile was verified by gas chromatography-mass spectrometry (GC-MS) measurements (Agilent Technologies, 6890N Network GC System, 5973 Network Mass Selective Detector). The final pH was checked (SevenEasy pH-meter, Mettler Toledo) and found to be 7.4. The samples were then stirred using a Teflon coated micro stir bar for 4 days at 25 °C. Transmission Electron Microscopy was used to confirm the formation of PAO (Fig. 1a). Aliquots were used for the AFM experiments. PAO were fibrillated adding a 5 v/v% hexane solution to the PAO solution, the resulting opalescent suspension was mixed in vortex (Advanced Vortex Mixer, VELP Scientifica) for 10 minutes and hexane was removed from the suspension muttering argon for 10 minutes. The complete removal of hexane was verified by GC-MS measurements.

### Mica surfaces preparation

Several mica slices of 5 × 20 × 0.5 mm were prepared cutting mica sheet by hand; each slice was carefully exfoliated before PAO or fibril adsorption to obtain a fresh and renewed plane surface, suited for AFM measurements.

### PAO and fibrils adsorption on mica surface (primary)

Slices of mica were soaked in a glass tube containing a PBS solution and PAO or fibrils (both deriving from 44 μM Aβ_42_ monomer solutions) for the different times of the experimental conditions. After the due time, the slices of mica were pulled out, washed with ddH_2_O and gently blow-dried with argon.

### Exposure of a secondary bare mica surface to primary surface containing PAO or fibrils

Experiments were conducted approaching a secondary bare mica surface to a primary (coated with PAO or fibrils) in a glass tube containing the buffer solution. In the investigated systems, the two facing sheets of mica were either separated by just the PBS solution film (contact), or by a 0.1 mm thick Teflon wire placed between the two surfaces in a loop shape to maintain constant the distance between the two surfaces (semi-contact).

Different experiments were conducted varying the composition of the medium between the two facing surfaces: PBS solution alone, PBS solution containing antibodies effective in recognizing amyloid fibers (OC)^2^ or Aβ_42_ PAO (VIA)^3^ (generous gift of Prof. Kayed, UTMD School of Medicine). Typical antibodies concentrations were approximately 5 μM. In the experiments, different contact times were used and both primary and secondary mica surfaces were analyzed.

### AFM instrument, calibration and experimental conditions

The AFM analysis was performed using a VEECO NanoScope IIIa equipped with TAP 150Al-G probe from Budget Sensors; the image collection was managed via the software NANOSCOPE IMAGE 5.31R1. All the surfaces were morphologically characterized in tapping mode: only bare mica was scanned on contact mode. As our main goal was to observe the structure of fibrils and oligomers rather than characterize their atomic structural organization, we preferred working in tapping mode with respect to contact mode to avoid tearing or deformation of the sample due to shear forces and any damage of the sample due to scraping by the tip. The surfaces of samples were analyzed by non-filtered 512 pixel/line images. Nonlinear relationship is corrected during the calibration routine by applying a non linear voltage in real-time to produce a linear scan in X and Y in both trace and retrace scan directions using a 10 μm pitch calibration grating in which the depth of each square is 200 nm.

After aligning the laser we set the vertical deflection signal of the photo detector to a value close to 0.0 Volts as starting value. To be sure that cantilever will vibrate at an appropriate amplitude (*i.e*., resonance peak is properly fixed), we tuned the cantilever before each measurement. We used the auto-tune function of the controlling software to determine the frequency that produces the largest response (resonance frequency).

The proper experimental parameters to be used during AFM measurements may vary significantly from point to point of the sample surface. For this reason, the scanning of each region starts from predetermined values, which are then adjusted during measurement. Three primary feedback parameters were adjusted every time the microscope is engaged to capture an image: integral and proportional gains, scan rate, and amplitude set point. For scan sizes up to 1 μm we fixed the integral gain to 0.4 and the scan rate to 2 Hz as initial scan parameters; at the beginning, the amplitude setpoint is automatically set by the software when running auto-tune or before engagement if auto-tune is not done. To keep a minimum force between the tip and sample, the amplitude setpoint was gradually optimized increasing its value until the tip lifts off the surface and then decreasing it a little. The scan rate was adjusted to a speed adequate to the dimensions of the surface features we wanted to analyze; wider scanned surface means lower scan rate values (e.g., 2 Hz for scan sizes of 1–4 μm). We then increased the integral and proportional gains up to the value beyond which the noise will increase as the feedback loop starts to oscillate and then we reduced the gains until the noise goes away. Quantitative analysis of the mica surfaces was performed on 4–6 images obtained by two different equivalent surfaces. Surface structures with anomalous shape or dimension were omitted. Heights are estimated by tracing profiles on the height signal images (GWYDDION software, v. 2.34).

#### Transmission electron microscopy

5 μl of the PAO suspension was adsorbed for 1 minute to a formvar/carbon coated grid. Excess liquid was removed with filter paper (Whatman #1) and the samples were stained with 1% uranyl acetate or 0.75% uranyl formate for 30 seconds. After removing the excess uranyl solution with a filter paper the grids were examined in a JEOL 1200EX Transmission Electron Microscope and images were recorded with an AMT 2 k CCD camera.

#### Limitations and future directions

We acknowledge the limitations of the *in-vitro* study to address biological processes. We acknowledge that since PAO are unstable and are in constant equilibrium with monomers, dimer-trimers and fibrils^24^,^25^,^26^ our *in-vitro* PAO preparation contains a mixed population of monomers, dimer-trimers and PAO as verified by size exclusion chromatography (data not shown). However, following adsorption of PAO and fibrils on mica, the substrates were dried and the structural features of the adsorbed species remained unchanged until they were put in contact with solution. Once in solution the structures begin to change and that is one of the goals of the present study. We also acknowledge that the hexane-induced fibril formation is not indicative of a physiological condition and the fibrils may have subtle differences in underlying structure *vs.* other methods of generating fibers^43^. However among the known method to generate *in vitro* amyloid fibers was the one with the least effect on our experimental setting. We chose a reductionist approach to begin dissecting the basis for the complex behavior of the dynamic changes of misfolded proteins in a simplified system. The findings obtained from this study may help directing future approaches in biological settings. We designed future studies to progressively introduce the known variables playing a role in the complex *in-vivo* system to test the mechanisms of PAO metastasis and toxicity to ultimately design targeted interventions.

## Additional Information

**How to cite this article**: Bernini, F. *et al*. Pre-amyloid oligomers budding: a metastatic mechanism of proteotoxicity. *Sci. Rep.* **6**, 35865; doi: 10.1038/srep35865 (2016).

## Supplementary Material

Supplementary Information
