# Corrigendum: Pre-amyloid oligomers budding:a metastatic mechanism of proteotoxicity

**DOI:** 10.1038/srep40897

**Published:** 2017-01-19

**Authors:** Fabrizio Bernini, Daniele Malferrari, Marcello Pignataro, Carlo Augusto Bortolotti, Giulia Di Rocco, Lidia Lancellotti, Maria Franca Brigatti, Rakez Kayed, Marco Borsari, Federica del Monte, Elena Castellini

Scientific Reports 6: Article number: 35865; 10.1038/srep35865 published online: 10 24 2016; updated: 01 19 2017.

The Author Contributions statement in this Article is incomplete:

F.B., D.M., M.P., L.L., F.d.M. and E.C. acquired and analyzed the data; C.A.B., G.D.R., M.F.B. and M.B. provided critical input to the analyzed data and to the manuscript; R.K. provided the structural antibodies and critical input to the manuscript; F.d.M. conceived the project; F.d.M. and E.C. designed the experiments, protocols and drafted the manuscript.

Should read:

F.B., D.M., M.P., L.L., F.d.M. and E.C. acquired and analyzed the data; C.A.B., G.D.R., M.F.B. and M.B. provided critical input to the analyzed data and to the manuscript; R.K. provided the structural antibodies and critical input to the manuscript; F.d.M. conceived the project; F.d.M. and E.C. designed the experiments, protocols and drafted the manuscript. F.B. and D.M. contributed equally to this work. F.d.M. and E.C. contributed equally to this work.

