# Supplementary material for: Pre-amyloid oligomers budding:a metastatic mechanism of proteotoxicity
Source: Sci Rep. 2016 Oct 24;6:35865. doi: 10.1038/srep35865 (PMC5075897; doi:10.1038/srep35865)
Supplement: Supplementary Information [file srep35865-s1.doc]

**Pre-amyloid oligomers budding:**

**a metastatic mechanism of proteotoxicity**

Fabrizio Bernini BS^1^, Daniele Malferrari PhD^1^, Marcello Pignataro BS^1,2^, Carlo Augusto Bortolotti PhD^1^, Giulia Di Rocco PhD^1^, Lidia Lancellotti BS^1^, Maria Franca Brigatti PhD^1^, Rakez Kayed, PhD^3^, Marco Borsari, PhD^1^, Federica del Monte MD, PhD^*2^, Elena Castellini PhD^*1^.

**Supplementary Figures**

**Supplementary Figure 1**: Schematic representation of the nucleation-dependent maturation of misfolded proteins. The process of amyloid fibers formation reduces the free energy of the system thus the aggregates acquire a thermodynamically more favorable conformation.

**Supplementary Figure 2**: Schematic representation of the experimental conditions for the exposure of secondary (IIry) mica surface to a primary (Iry) one, coated by PAO or fibrils.

**Supplementary Figure 3.** TM topography images of OC (**a**) and VIA (**b**) antibodies adsorbed on bare mica by dipping the surface for 1hr in a 5 µM OC or VIA antibodies solution in 10mM phosphate buffer at pH 7.4, T = 25°C.

Supplementary Figure 1


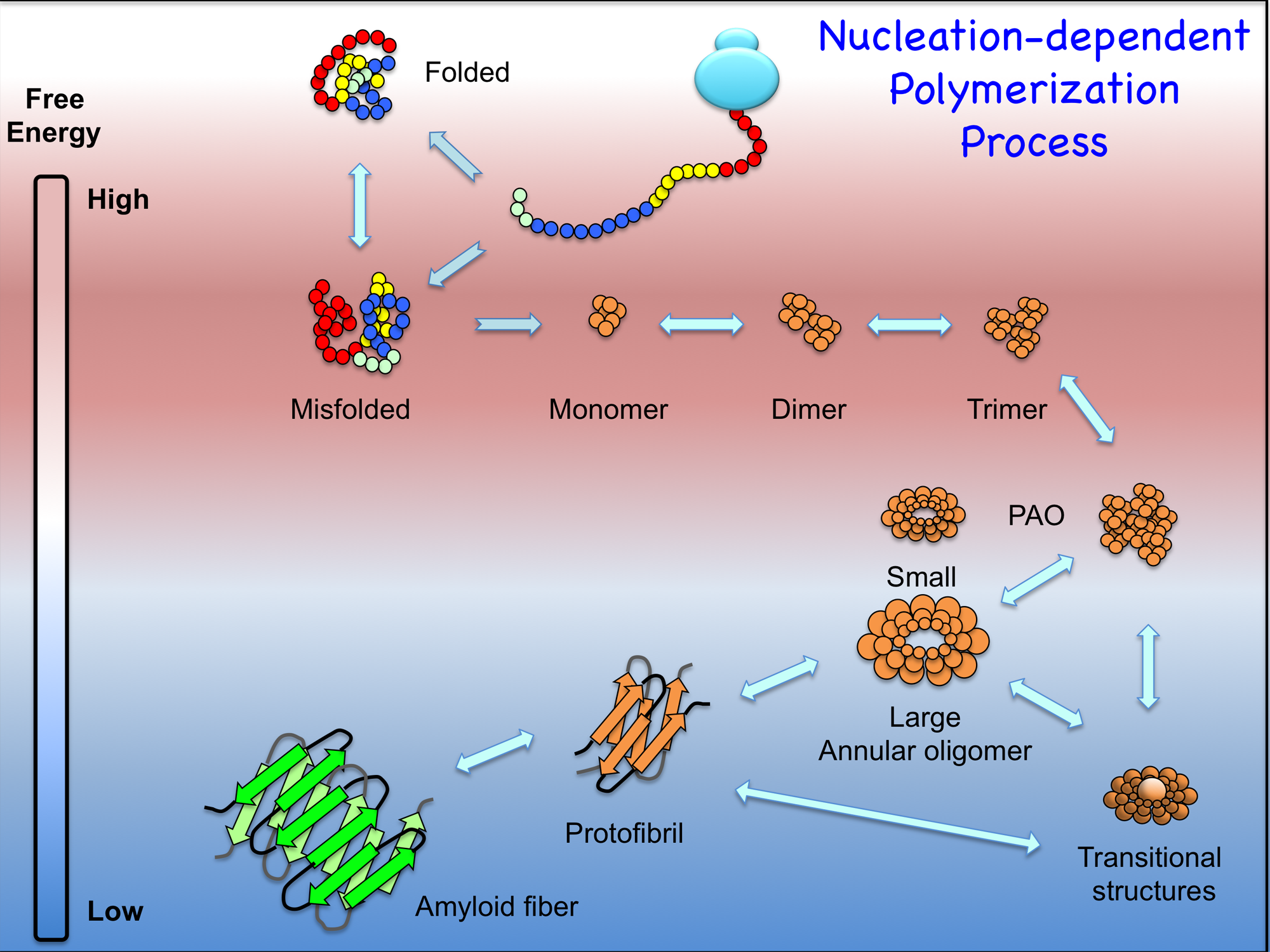


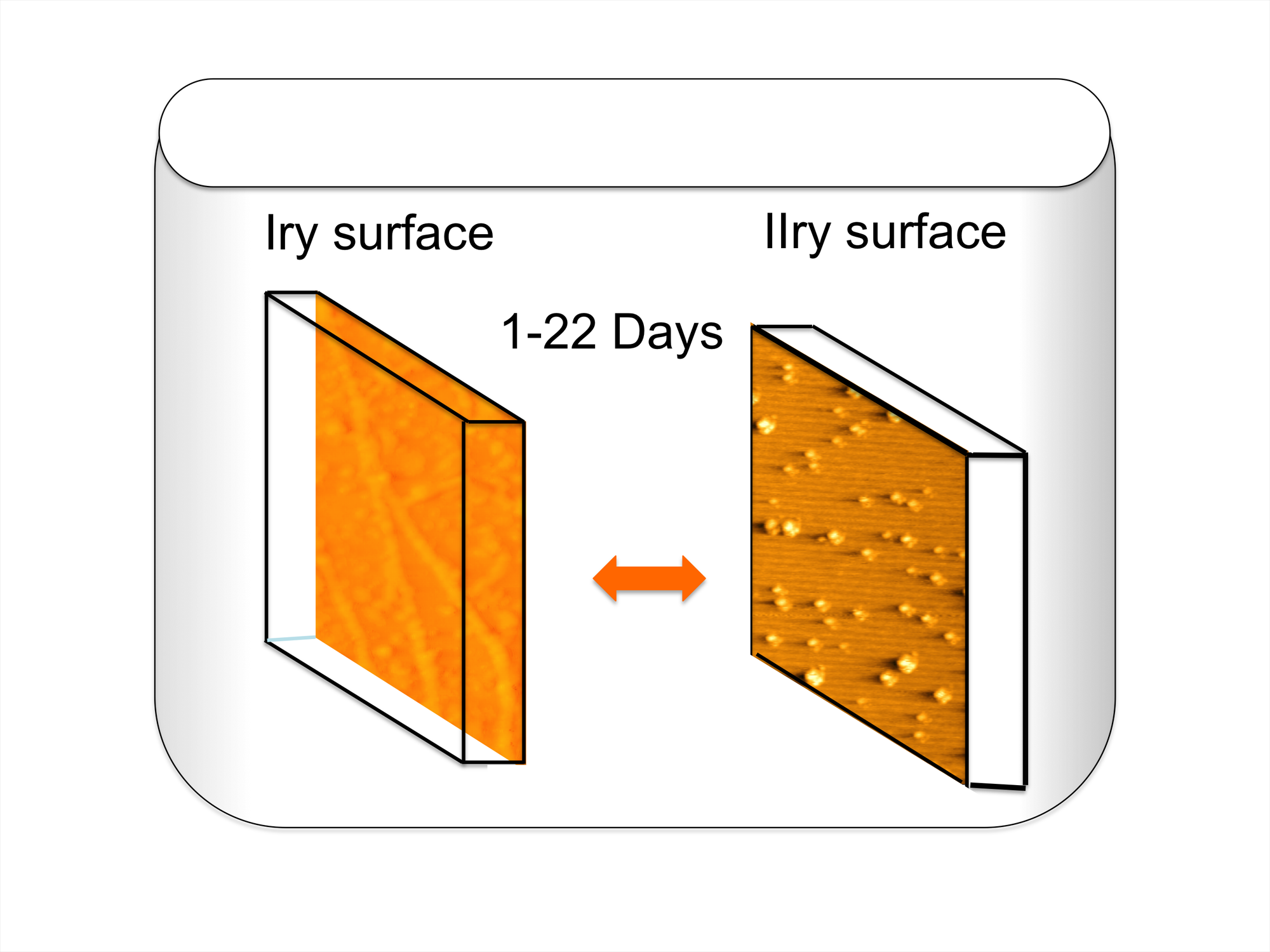


Supplementary Figure 2


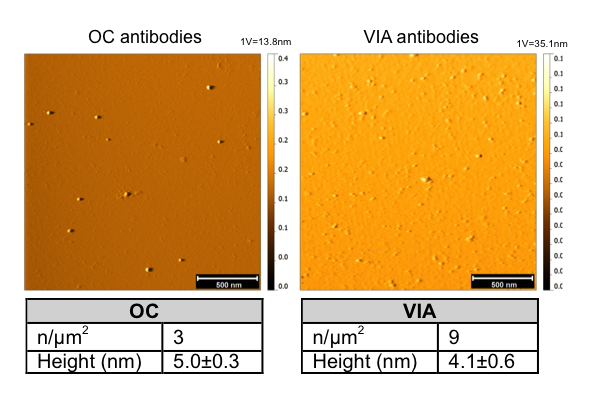


Supplementary Figure 3
